# Effects of roads on *Castanopsis carlesii* seedlings and their leaf herbivory in a subtropical forest in China

**DOI:** 10.1093/jis/14.1.17

**Published:** 2014-01-01

**Authors:** Xiao-Hua Dai, Jia-Sheng Xu, Lu-Rong Cai

**Affiliations:** 1 School of Life and Environmental Sciences, GanNan Normal University, Ganzhou 341000, China; 2 National Navel-Orange Engineering Research Center, Ganzhou 341000, China

**Keywords:** leaf miners, leaf gallers, road ecology, species diversity

## Abstract

The effects of a forest road on *Castanopsis carlesii* (Hemsley) Hayata (Fagales: Fagaceae) seedlings and their leaf herbivory were investigated in a subtropical forest at Jiulianshan National Nature Reserve, Jiangxi, China. A total of 1124 seedlings, 33949 leaves, 468 leaf mines, and 205 leaf galls were found. Generally, individual numbers, tree heights, and leaf numbers of *C. carlesii* seedlings became lower with increasing distances from the road. These results might indicate that old seedlings were fewer and survival rate of seedlings was lower in forest interiors. Leaf miners preferred the seedlings close to the forest road, while leaf gallers preferred the seedlings about 2 m from the road. Species diversity of leaf miners was higher in the forest interior area, while species diversity of leaf gallers was higher near the road. However, both leaf miners and leaf gallers decreased in general from the road to the interior forest. There were interspecific differences in the effects of roads on leaf miner species and leaf galler species. The effects of the road on seedlings and insects could be explained by varying microhabitat conditions and different ecological strategies.

摘要

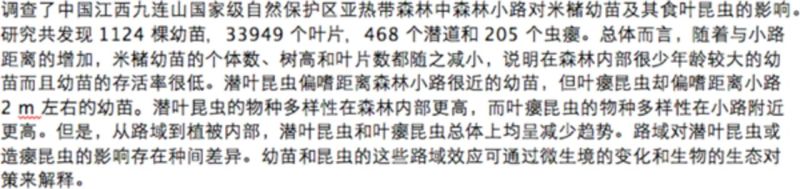

## Introduction

Roads have large ecological effects on plants and insects. First, a road can change atmospheric and soil conditions. Microhabitat conditions such as pollutants, light, temperature, moisture, and nutrients vary between locations near roads vs. far from roads ( Spencer and Port 1988 ; Spencer et al. 1988 ; Angold 1997 ; Avon et al. 2010 ).

With the above microhabitat variations at different distances from roads, plant characteristics will change accordingly. Generally, litter depth, canopy cover, canopy height, basal area, population density, total biomass, species diversity of native plants, shade-requiring plants, and geophytes are smaller at road edges ( Watkins et al. 2003 ; Godefroid and Koedam 2004 ; Flory and Clay 2009 ; Avon et al. 2010 ), while understory cover, species diversity of exotic plants, gramnoids, heliphilous plants, disturbance species, and therophytes are higher when adjacent to the roads ( Watkins et al. 2003 ; Godefroid and Koedam 2004 ; Avon et al. 2010 ). Plant individuals have higher body size, biomass, dry weight, nitrogen, water potential, lead, sodium, manganese, leaf number, flower number, shoot length, and shoot diameter on road edges ( Spencer and Port 1988 ; Spencer et al. 1988 ; Lightfoot and Whitford 1991 ; Martinez and Wool 2006 ), but individuals have lower resin and sulfate near the roadside ( Lightfoot and Whitford 1991 ). Native plants germinate, survive, and grow better away from roads ( Martel 1995 ; Comita and Goldsmith 2008 ), while exotic plants perform better near roads ( Flory and Clay 2009 ). However, plants of different species or different ages may show different responses to road-distance effects ( Angold 1997 ; Flory and Clay 2006 ; Avon et al. 2010 ).

Finally, along with effects of roads on atmosphere, soil, and plants, animals will distribute and behave differently at different distances from roads. For vertebrates and marcoinverte-brates, abundance, diversity, density, pairing success, reproduction, survival, and path tor-tuosity become higher away from roads, while territory size, dispersal distance, mortality, and lead concentration become lower away from roads ( Reijnen et al. 1995 ; Ortega and Capen 2002 ; Marsh and Beckman 2004 ). For insects, abundance, diversity, density, development time, reproduction, and fecundity become lower away from roadside regions ( Muskett and Jones 1980 ; Spencer and Port 1988 ; Lightfoot and Whitford 1991 ; Koivula 2005 ; Martinez and Wool 2006 ; Dunn and Danoff-Burg 2007 ). However, animals of different species, different sexes, and different ages may respond differently to roads ( Reijnen et al. 1995 ; Ortega and Capen 2002 ; Marsh and Beckman 2004 ; Martinez and Wool 2006 ). Distance effects of roads on plants, insects, and animals may vary with different scales and with different road types ( Forman and Alexander 1998 ; Dunn and Da-noff-Burg 2007 ; Comita and Goldsmith 2008 ; Avon et al. 2010 ).

*Castanopsis carlesii* (Hemsley) Hayata (Fagales: Fagaceae) is a dominant tree species in the subropical evergreen broadleaf forest in South China. In natural habitats, seedling banks of *C. carlesii* are abundant in forest understory. Moreover, plenty of insect herbivory patterns, including leaf mines and leaf galls, are found on *C. carlesii* adults and seedlings ( Wang et al. 2006 ). Mines and leaf galls are distinctive marks of endophagous organisms on host plants ( Krassilov 2008 ). Most miners and gallers are monophagous or oligophagous, while structures of many mines and galls are speciesspecific or genus-specific ( Dai et al. 2011 ). Since the types and numbers of leaf mines and leaf galls can be easily identified, mines and galls are ideal for studies on road effects.

In this study, small-scale effects of a forest road on leaf herbivory of *Castanopsis carlesii* seedlings in a subtropical forest were quantified. The following two questions were addressed: 1) Were there different responses by plant seedlings, leaf miners, and leaf gallers to roads? 2) Were there interspecific differences in the effects of roads on leaf miner species leaf galler species?

## Materials and Methods

### Study site

Jiulianshan National Nature Reserve (24° 29’ 18”–24° 38’ 55” N, 114° 22’ 50”–114° 31’ 32” E) is located in the south of Jiangxi Province, China. It covers 134 km ^2^ with an altitude range from 280 to 1,434 m a.s.l. The climate is subtropical, and the annual precipitation is 2,156 mm. The mean monthly temperature ranges from 6.8°C (January) to 24.4°C (July). The dominant vegetation is subtropical evergreen broadleaf forest, low hill coniferous forest, bamboo forest, montane dwarf forest, and montane grassland ( Liu et al. 2002 ). *C. carlesii* is one dominant tree species in the area.

### Data collection

**Figure 1.**
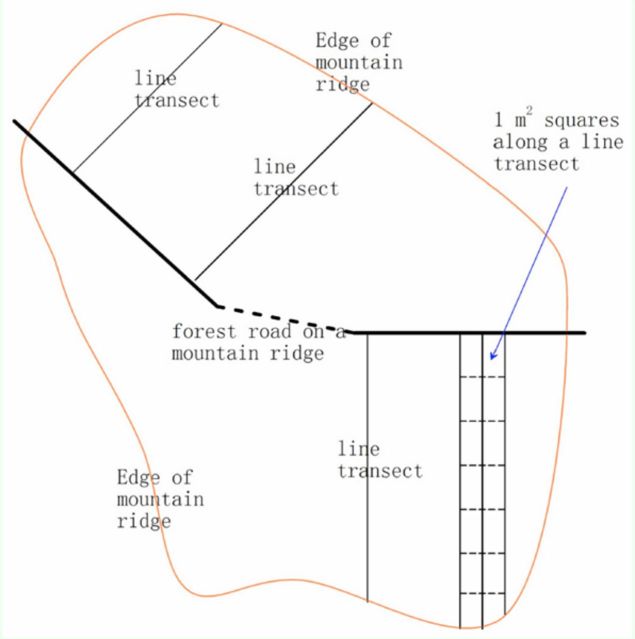
Line transects (thin black line) were perpendicular to the forest road (thick black line, solid or dotted) on the mountain ridge. Seedlings and their herbivory (mines and galls) were investigated in 1 m2 areas along each line transect. Note that it is a non-scale schematic diagram, not a real map of the investigation site and line transects. High quality figures are available online.

The seedlings of *C. carlesii* mainly occurred in forest edges. A mountain ridge was chosen because there were plenty of seedlings ( Figure 1 ). Along a small forest road under the forest cover, multiple line transects were set perpendicular to the road. The distances between two transects was 5 m. A total of 19 transects on one side of the road and nine transects on the other side were sampled. All seedlings inside ±0.5 m to each transect were investigated. Each transect was divided 1 m after 1 m from the road so a series of 1 m ^2^ areas along one transect could be therefore obtained. Generally, the number of complete squares along one transect was fewer than six ( Figure 1 ). Distance classes were set according to the midpoint of each square, that is, 0.5 m, 1.5 m, 2.5 m, etc. The last square for each transect wasn’t used, since it was usually incomplete. The identification of leaf mines and leaf galls were based on mine or gall characteristics. In previous studies, some leafmining species had been reared and identified ( Dai et al. 2013 ).

The number of seedling individuals, number of seedling leaves, accumulated tree height, number of mines per leaf, number of galls per leaf, number of each leaf mine type, and number of each leaf gall type were calculated for all seedlings in each complete square. Mine diversity and gall diversity were measured at each distance class by both Simpson’s diversity index ( *D* ) and Shannon’s diversity index ( *H* ).

### Statistical analysis

Generalized additive models (GAM) were used to describe plant and herbivory parameters in relation to distance from the road. GAM approach was used because no *a priori* assumptions should be made about the relationship ( Hastie and Tibshirani 1990 ; Rokaya et al. 2012 ). GAM fitting with a smoothing spline was performed with PAST version 2.15 ( Hammer et al. 2001 ). All data points at the same distance class were collapsed to a single point by weighted averaging and calculation of a combined standard deviation. Since standard deviation was unspecified for *D* or *H* , it was set to the default 10% of the standard deviation of *D* or *H* in PAST program. Optimal smoothing was calculated by a cross-validation procedure. The optimal smoothing factor of De Boor (2001) was thus obtained to fit a third-order polynomial spline.

## Results

A total of 1,124 seedlings, 33,949 leaves, 468 leaf mines, 205 leaf galls, four leaf mine types (i.e., LM01, LM03, LM06 and LM09), and five leaf gall types (i.e., LG01, LG03, LG04, LG05 and LG06) were found in the investigation. The leaf miner species of LM01, LM03, LM06 were *Stigmella* sp. (Lepidoptera: Nepticulidae), *Tischeria* sp. (Tischeriidae), and *Acrocercops* sp. (Gracillariidae). LM09 and leaf gall species were not identified.

**Figure 2.**
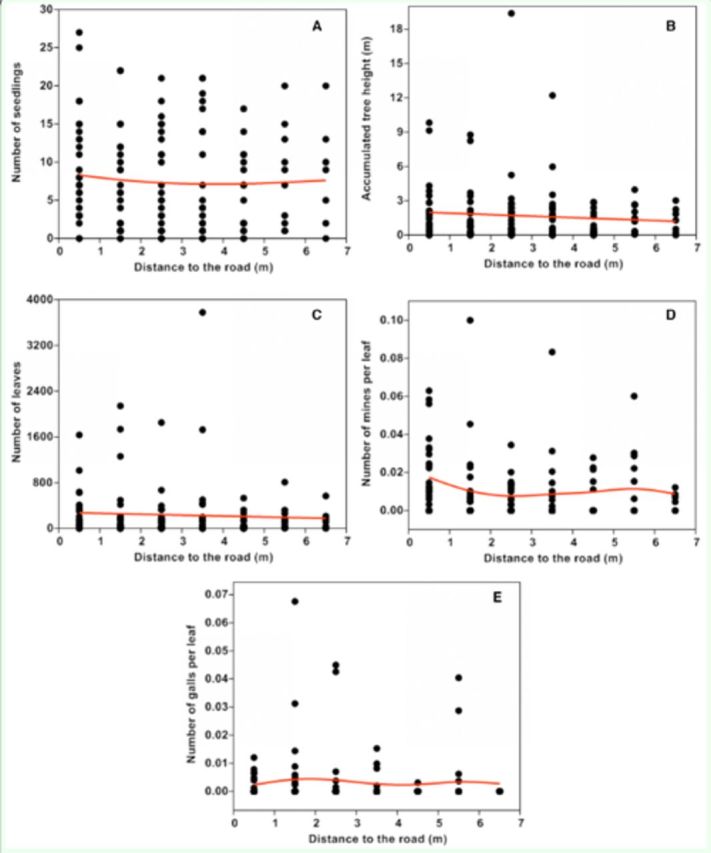
Scatterplots of number of seedlings (a), accumulated tree height (b), number of leaves (c), number of mines per leaf (d), and number of galls per leaf (e) against distance from road. The line was fitted by generalized additive models with a third-order polynomial smoothing spline. High quality figures are available online.

Numbers of *C. carlesii* seedlings in each 1-m ^2^ area were highest 0.5 m and 6.5 m from the road but lowest 3.5 m from the road; however, if a subset of squares with large seedling numbers only were considered, seedling numbers seemed to drop with the increasing distances ( Figure 2a ). Accumulated tree heights and numbers of leaves and of *C. carlesii* seedlings in each square decreased linearly against the distances from the road. Considering larger values of tree heights and leaf numbers only, the trends were also similar ( Figure 2b , c ).

The number of mines or galls in each seedling was linearly correlated with the number of leaves of the seedling (r = 0.518, *P* < 0.001; r = 0.539, *P* < 0.001 respectively). Therefore, the number of mines or galls per leaf should be used for the measurement of herbivory.

The number of mines per leaf in each square peaked at 0.5 m and dropped fluctuantly. When only large mine number values were considered, a decreasing trend could be noticed ( Figure 2d ). The number of galls per leaf in each square first peaked at 2 m and peaked again 5.5 m from road, a pattern that was consistent with that of large gall number values ( Figure 2e ).

**Figure 3.**
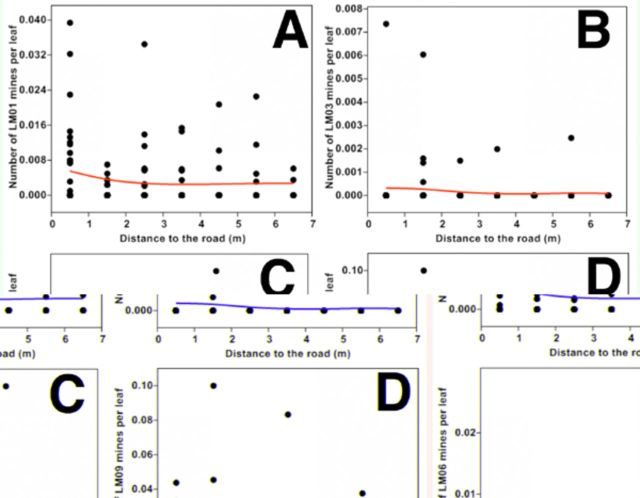
Scatterplots of number of different leaf mine types per leaf against distance from road: LM01 (a), LM03 (b), LM06 (c), and LM09 (d). The line was fitted by generalized additive models with a third-order polynomial smoothing spline. High quality figures are available online.

For different leaf miners, numbers of LM01, LM03, and LM09 per leaf peaked 0.5 m from the road and then dropped. The number of LM06 per leaf was very low at all distance classes and increased linearly but slowly against the distances from the road. Patterns of every leaf mine type were consistent to the variations of their large values ( Figure 3 ).

**Figure 4.**
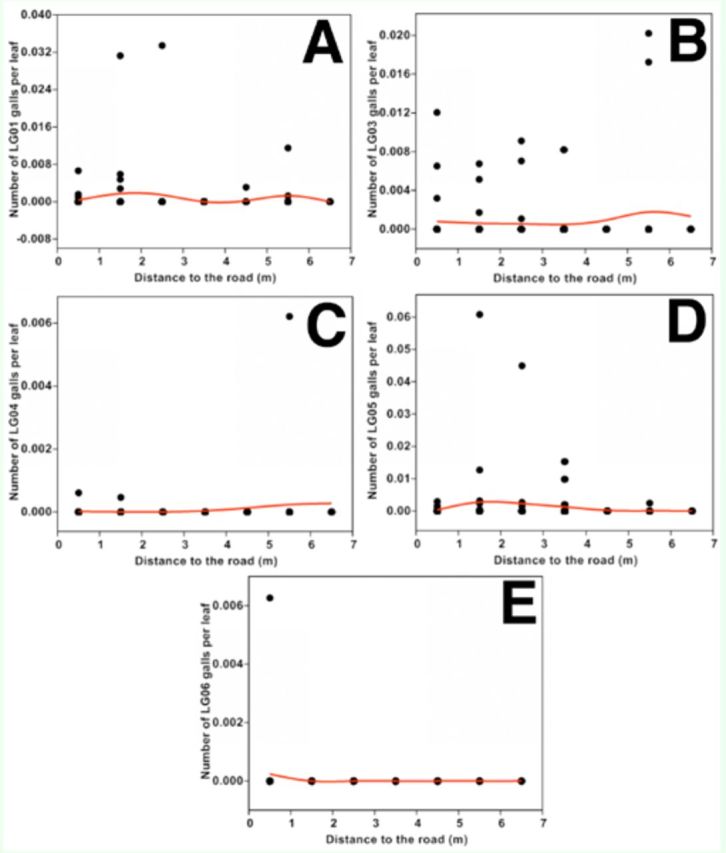
Scatterplots of number of different leaf gall types per leaf against distance from road: LG01 (a), LG03 (b), LG04 (c), LG05 (d), and LG06 (e). The line was fitted by generalized additive models with a third-order polynomial smoothing spline. High quality figures are available online.

For different leaf gallers, the number of LG01 per leaf peaked at about 2 m and 5.5 m from the road, which was similar to the pattern of total gall numbers. The number of LG03 per leaf peaked 5.5 m from the road. The number of LG04 and LG06 per leaf was very low at all distance classes, but LG04 numbers increased after 3.5 m from road, and LG06 numbers dropped from 0.5 m from road. The number of LG05 per leaf peaked about 1.5 m from the road and then dropped. Patterns of every leaf gall type were consistent to the variations of their large values ( Figure 4 ).

**Figure 5.**
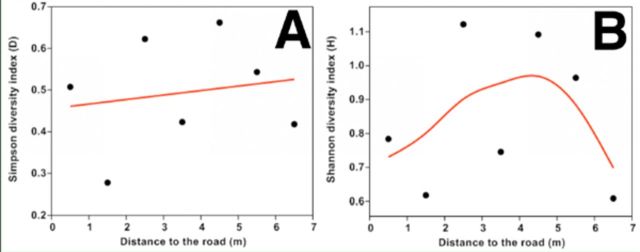
Relationship between leaf mine diversity with distance from road: Simpson’s diversity *D* (a) and Shannon’s diversity *H* (b). The line was fitted by generalized additive models with a third-order polynomial smoothing spline. High quality figures are available online.

**Figure 6.**
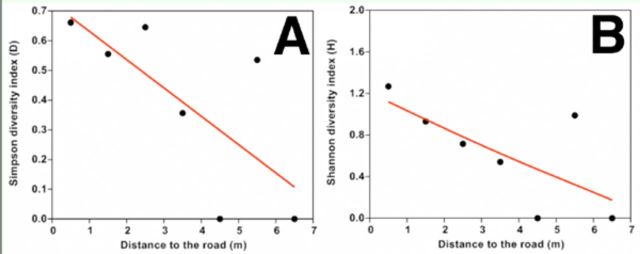
Relationship between leaf gall diversity with distance from road: Simpson’s diversity *D* (a) and Shannon’s diversity *H* (b). The line was fitted by generalized additive models with a third-order polynomial smoothing spline. High quality figures are available online.

Both Simpson’s diversity index ( *D* ) and Shannon’s diversity index ( *H* ) of leaf miners increased from 0.5 m to 4.5 m from road, and then *H* dropped ( Figure 5 ), while *D* and *H* of leaf gallers decreased continuously with increasing distance from road ( Figure 6 ).

Generally, the density of different leaf mines was ordered as follows: LM09 > LM01 > LM03 > LM06 ( Figure 3 ); the density of different leaf galls was ordered as follows: LG05 > LG01 ≈ LG03 > LG04 ≈ LG06 ( Figure 4 ).

## Discussion

Generally, the number of individuals, tree heights, and the number of leaves of *C. carlesii* seedlings became lower with increased distance from road. These results might indicate that old seedlings were fewer and the survival rate of seedlings was lower in forest interiors ( Comita and Goldsmith 2008 ).

Leaf miners and leaf gallers on *C. carlesii* seedlings showed different responses to the distance from the road. For example, leaf miners preferred the seedlings close to the forest road while leaf gallers preferred the seedlings about 2 m away from the road. It is also interesting that species diversity of leaf miners was higher in the forest interior while species diversity of leaf gallers was higher near the road. However, from the edge of the road to interior vegetation, both leaf miners and leaf gallers generally decreased. Such patterns have been found for isopods, hemipterans, collembolans, sap-feeding herbivores, carabids, and carrion beetles ( Muskett and Jones 1980 ; Lightfoot and Whitford 1991 ; Koivula 2005 ; Dunn and Danoff-Burg 2007 ).

For leaf miners, the number-distance patterns of LM01 ( *Stigmella* sp.), LM03 ( *Tischeria* sp.) and LM09 (an unknown leafminer) were similar to those of total leaf mines. However, the variations of LM03 at different distances were not so obvious. Contrarily, LM06 (another Gracillariid) only occurred in the forest interior ( Figure 3 ). Seedlings suffered different damage levels from different leaf miners. There existed interspecific differences of the effects of the road among leaf miner species.

For leaf gallers, seedlings endured different damage levels from different leaf gallers. The influence of the road on LG01, LG03, LG04, LG05, and LG06 was different from species to species. Such interspecific differences between galling insects were also discovered in other studies ( Martel 1995 ; Martinez and Wool 2006 ).

The ecological strategies of plants or animals are related to their distribution and behavior in accordance to the roads ( Koivula 2005 ; Martinez and Wool 2006; Flory and Clay 2009 ; Avon et al. 2010 ). For example, insect species that prefer vigorous plants may choose roadside plants ( Lightfoot and Whitford 1991 ; Price 1991 ) while insects using stressed plants may use plants under the vegetation cover ( Martel 1995 ; Martinez and Wool 2006 ). Such theories might help to explain the varying effects of the road on different insect species in this study.

Since *C* . *carlesii* adults were dominant in the first tree layers, they must prefer the sun environment. Their seedlings might also grow better in more sunny conditions near the roads. According to another investigation on the distribution of leaf herbivory in a different canopy area of four Fagaceae tree species, including *C* . *carlesii* , in the same nature reserve, both leaf miners and leaf gallers generally preferred sun leaves rather than shade leaves, while leaf gallers had a lower degree of preference to sun than leafminers ( Dai et al. 2013 ). These phenomena are consistent with this study: leaf mines were most abundant on leaves that were fully in the sun near roads, but leaf galls were not abundant on leaves that were fully in the sun, mostly occurring on leaves exposed to moderate sun a little father away from the roads. For specific species, the mines of LM01 ( *Stigmella* sp.) were mostly found in the leaves in the sun near the road, which is consistent with our previous observations ( Dai et al. 2013 ) but contrary to other *Stigmella* ( Nielsen and Ejlersen 1977 ; van Nieukerken et al. 2006 ). The mines of LM03 ( *Tischeria* sp.) were more numerous in roadside sun leaves, but the trend was not distinct, which is similar to our other investigation ( Dai et al. 2013 ). However, a *Tischeria* sp. has no perferences between sun leaves or shade leaves of *Quercus emoryi* ( Bultman and Faeth 1988 ; Connor 2006 ). The mines of LM09 (an unknown leafminer) occurred only in young leaves, which were more in roadside plant individuals. Some gracillariid moths also preferred shade leaves ( Nielsen and Ejlersen 1977 ; Faeth 1991a ; Faeth 1991b ; Angulo-Sandoval and Aide 2000 ), just like LM06 (another Gracillariid miner).
